# 4-(Piperidin-1-yl)-4*H*-benzo[*b*]tetra­zolo[1,5-*d*][1,4]diazepin-5(6*H*)-one

**DOI:** 10.1107/S1600536810049950

**Published:** 2010-12-04

**Authors:** Gary S. Nichol, Zhigang Xu, Christine E. Kaiser, Christopher Hulme

**Affiliations:** a Department of Chemistry and Biochemistry, 1306 E University Boulevard, The University of Arizona, Tucson, AZ 85721, USA; b Southwest Center for Drug, Discovery and Development, College of Pharmacy, BIO5 Institute, The University of Arizona, Tucson, AZ 85721, USA

## Abstract

There are two crystallographically unique mol­ecules present in the asymmetric unit of the title compound, C_14_H_16_N_6_O; in both mol­ecules, the seven-membered diazepinone ring adopts a boat-like conformation and the chair conformation piperidine ring is an axial substituent on the diazepinone ring. In the crystal, each mol­ecule forms hydrogen bonds with its respective symmetry equivalents. Hydrogen bonding between mol­ecule *A* and symmetry equivalents forms two ring motifs, the first formed by inversion-related N—H⋯O inter­actions and the second formed by C—H⋯O and C—H⋯N inter­actions. The combination of both ring motifs results in the formation of an infinite double tape, which propagates in the *a*-axis direction. Hydrogen bonding between mol­ecule *B* and symmetry equivalents forms one ring motif by inversion-related N—H⋯O inter­actions and a second ring motif by C—H⋯O inter­actions, which propagate as a single tape parallel with the *c* axis.

## Related literature

The structure of the title compound was determined as part of a larger study on development of synthetic methods for high-throughput medicinal chemistry. For background to the use of multi-component reactions in high-throughput medicinal chemistry, see: Gunawan *et al.* (2010 ▶); Hulme & Dietrich (2009 ▶); Hulme & Gore (2003 ▶). For the Ugi reaction, see: Ugi & Steinbrückner (1961 ▶). For graph-set notation for hydrogen bonding, see: Bernstein *et al.* (1995 ▶) and puckering parameters, see: Cremer & Pople (1975 ▶).
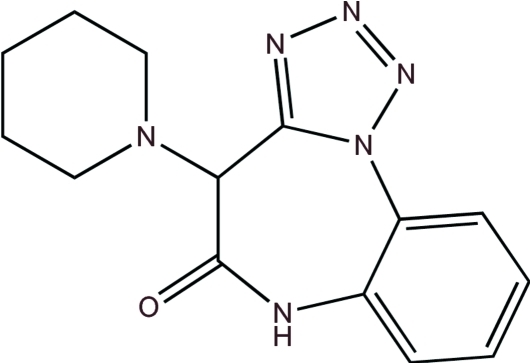


## Experimental

### 

#### Crystal data

C_14_H_16_N_6_O
*M* *_r_* = 284.33
Triclinic, 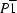

*a* = 8.8210 (7) Å
*b* = 13.1802 (10) Å
*c* = 13.4476 (11) Å
α = 105.549 (2)°
β = 99.490 (2)°
γ = 106.623 (2)°
*V* = 1392.99 (19) Å^3^
*Z* = 4
Mo *K*α radiation
μ = 0.09 mm^−1^
*T* = 100 K
0.39 × 0.28 × 0.09 mm

#### Data collection

Bruker Kappa APEXII DUO CCD diffractometer
Absorption correction: numerical (*SADABS*; Sheldrick, 1996 ▶) *T*_min_ = 0.965, *T*_max_ = 0.992
51078 measured reflections
12177 independent reflections
9733 reflections with *I* > 2σ(*I*)
*R*_int_ = 0.029

#### Refinement

*R*[*F* ^2^ > 2σ(*F* ^2^)] = 0.043
*wR*(*F* ^2^) = 0.123
*S* = 1.05
12177 reflections
507 parameters
All H-atom parameters refined
Δρ_max_ = 0.59 e Å^−3^
Δρ_min_ = −0.23 e Å^−3^

### 

Data collection: *APEX2* (Bruker, 2007 ▶); cell refinement: *SAINT* (Bruker, 2007 ▶); data reduction: *SAINT*; program(s) used to solve structure: *SHELXTL* (Sheldrick, 2008 ▶); program(s) used to refine structure: *SHELXTL*; molecular graphics: *ORTEP-3 for Windows* (Farrugia, 1997 ▶) and *Mercury* (Macrae *et al.* 2008 ▶); software used to prepare material for publication: *SHELXTL* and local programs.

## Supplementary Material

Crystal structure: contains datablocks I, global. DOI: 10.1107/S1600536810049950/kj2167sup1.cif

Structure factors: contains datablocks I. DOI: 10.1107/S1600536810049950/kj2167Isup2.hkl

Additional supplementary materials:  crystallographic information; 3D view; checkCIF report

## Figures and Tables

**Table 1:** Hydrogen-bond geometry (Å, °)

*D*—H⋯*A*	*D*—H	H⋯*A*	*D*⋯*A*	*D*—H⋯*A*
N1—H1*N*⋯O1^i^	0.850 (14)	2.069 (14)	2.9089 (9)	169.4 (13)
N51—H51*N*⋯O51^ii^	0.886 (16)	1.929 (16)	2.8116 (10)	173.6 (14)
C6—H6⋯N2^iii^	0.936 (15)	2.531 (15)	3.4638 (11)	174.8 (12)
C7—H7⋯O1^iii^	0.947 (15)	2.406 (15)	3.3394 (10)	168.4 (13)
C55—H55⋯N54^iv^	0.974 (13)	2.548 (13)	3.2293 (11)	127.0 (10)

Symmetry codes: (i) 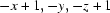; (ii) 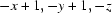; (iii) 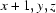; (iv) 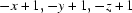.

**Fig. 1.**
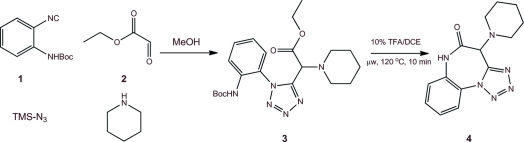
The synthetic route to 4.

**Fig. 2.**
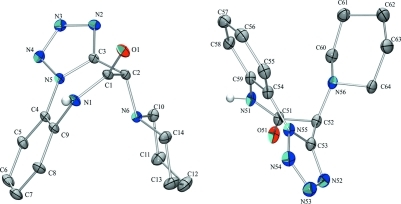
The asymmetric unit of 4 with displacement ellipsoids at the 50% probability level and C-bound H atoms omitted.

**Fig. 3.**
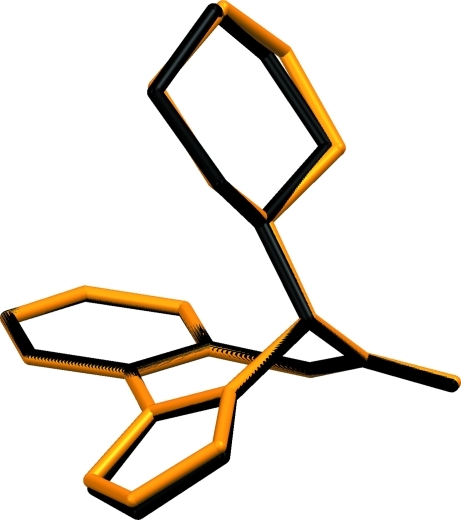
An overlay of molecule A (orange) and molecule B (black), r.m.s. deviation = 0.0185 Å), in 4.

**Fig. 4.**
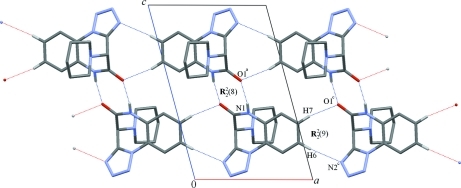
Hydrogen bonding patterns (dotted blue lines) formed by molecule A in 4. Symmetry operations: a, -x + 1, -y, -z + 1; c, x + 1, y, z.

**Fig. 5.**
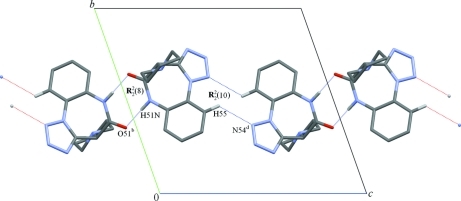
Hydrogen bonding patterns (dotted blue lines) formed by molecule B in 4. Symmetry operations: b, -x + 1, -y + 1, -z; d, -x + 1, -y + 1, -z + 1.

## supplementary crystallographic information

### Comment

At present there is a huge need for unique small molecules in the lead development stages of drug discovery. In this process, speed is paramount, and the development of high speed parallel synthesis in concert with isocyanide based multi-component reactions (MCRs) has enabled a revolution in high-throughput medicinal chemistry (Gunawan *et al.*, (2010); Hulme & Dietrich (2009); Hulme & Gore (2003)). Following this theme, a novel two step solution phase protocol for the synthesis of an array of tricyclic fused tetrazole-benzodiazepines was recently investigated (Figure 1). The methodology employs *ortho*-*N*-Boc benzylisonitriles 1 and ethyl glyoxylate 2 in the 4-component TMS-N_3_ modified Ugi reaction (Ugi & Steinbrückner, 1961) to assemble the desired product 3. Subsequent treatment with trifluoroacetic acid unmasks an internal amino nucleophile and promotes cyclization to form the diazepine ring of the generic structure 4. Here we report the crystal structure of 4.

The asymmetric unit of 4 is shown in Figure 2. There are two crystallographically unique molecules in the asymmetric unit; the molecule composed of atoms O1 to C14 will henceforth be referred to as "molecule A" and the molecule composed of atoms O51 to C64 referred to as "molecule B". Where appropriate, discussion will be limited to molecule A with results for molecule B presented in square brackets. Molecular dimensions are unexceptional.

The molecule adopts a U-shaped conformation in which the 7-membered diazepinone ring has adopted a boat-like conformation (total Q parameter 0.8021 (8)Å [0.8177 (9) Å]; Cremer & Pople (1975)) and the chair conformation piperidinyl ring is an axial substituent on the diazepinone ring. Both molecules have a very similar overall shape as shown by an overlay, fitting N1, N5, C4 > C9 with N51, N55, C54 > C59 (these representing the largest planar moiety in the structure, Figure 3).

In the crystal each molecule forms hydrogen bonds with its respective symmetry equivalents. Hydrogen bonding between molecule A and symmetry equivalents forms two ring motifs (Bernstein *et al.*, 1995), an *R*^2^_2_(8) motif formed by inversion-related N—H···O interactions and an *R*^2^_2_(9) motif formed by C—H···O and C—H···N interactions. The combination of both ring motifs results in the formation of an infinite double tape which propagates in the *a* axis direction (Figure 4). Hydrogen bonding between molecule B and symmetry equivalents forms one ring motif composed of an *R*^2^_2_(8) motif formed by inversion-related N—H···O interactions and an *R*^2^_2_(10) motif formed by C—H···O interactions (Figure 5). This propagates as a single tape parallel with the *c* axis.

### Experimental

A solution of piperidine (0.017 g, 0.20 mmol) and ethyl glyoxylate (0.04 ml, 50% in toluene, 0.20 mmol) in methanol (0.5 ml) were stirred at room temperature. After 5 minutes, *ortho*-*N*-Boc-phenylisonitrile (0.0436 g, 0.20 mmol) and trimethylsilylazide (0.023 g, 0.20 mmol) was added dropwise to the above solution and stirred at room temperature for 23 h. The solvent was evaporated *in vacuo* and the product was purified using column chromatography (5–30% Hexane/Ethyl Acetate) to afford the desired Ugi product (0.056 g, 0.20 mmol, 65%) as colorless oil. The purified Ugi product was treated with 10% trifluoroacetic acid in dichloroethane (4 ml) and irradiated in a Biotage Initiator&trade; for 10 minutes at 120°C. The organic layer was washed with 1*M* NaHCO_3_ (3 × 5 ml) and dried (MgSO_4_). The solvent was evaporated *in vacuo* and purified by column chromatography (0–50% Hexane/Ethyl Acetate) to afford the desired product (0.030 g, 0.116 mmol, 92%) as a white solid.

### Refinement

All hydrogen atoms were located in a difference Fourier map and are freely refined.

### Crystal data

**Table:**

C_14_H_16_N_6_O	*Z* = 4
*M**_r_* = 284.33	*F*(000) = 600
Triclinic, *P*1	*D*_x_ = 1.356 Mg m^−^^3^
Hall symbol: -P 1	Mo *K*α radiation, λ = 0.71073 Å
*a* = 8.8210 (7) Å	Cell parameters from 9970 reflections
*b* = 13.1802 (10) Å	θ = 2.5–35.5°
*c* = 13.4476 (11) Å	µ = 0.09 mm^−^^1^
α = 105.549 (2)°	*T* = 100 K
β = 99.490 (2)°	Prism, colourless
γ = 106.623 (2)°	0.39 × 0.28 × 0.09 mm
*V* = 1392.99 (19) Å^3^	

### Data collection

**Table:**

Bruker Kappa APEXII DUO CCD diffractometer	12177 independent reflections
Radiation source: fine-focus sealed tube with Miracol optics	9733 reflections with *I* > 2σ(*I*)
graphite	*R*_int_ = 0.029
φ and ω scans	θ_max_ = 35.0°, θ_min_ = 1.6°
Absorption correction: numerical (*SADABS*; Sheldrick, 1996)	*h* = −8→14
*T*_min_ = 0.965, *T*_max_ = 0.992	*k* = −21→21
51078 measured reflections	*l* = −21→19

### Refinement

**Table:**

Refinement on *F*^2^	Primary atom site location: structure-invariant direct methods
Least-squares matrix: full	Secondary atom site location: difference Fourier map
*R*[*F*^2^ > 2σ(*F*^2^)] = 0.043	Hydrogen site location: difference Fourier map
*wR*(*F*^2^) = 0.123	All H-atom parameters refined
*S* = 1.05	*w* = 1/[σ^2^(*F*_o_^2^) + (0.0711*P*)^2^ + 0.1991*P*] where *P* = (*F*_o_^2^ + 2*F*_c_^2^)/3
12177 reflections	(Δ/σ)_max_ < 0.001
507 parameters	Δρ_max_ = 0.59 e Å^−^^3^
0 restraints	Δρ_min_ = −0.23 e Å^−^^3^

### Special details

**Table:**

Geometry. All e.s.d.'s (except the e.s.d. in the dihedral angle between two l.s. planes) are estimated using the full covariance matrix. The cell e.s.d.'s are taken into account individually in the estimation of e.s.d.'s in distances, angles and torsion angles; correlations between e.s.d.'s in cell parameters are only used when they are defined by crystal symmetry. An approximate (isotropic) treatment of cell e.s.d.'s is used for estimating e.s.d.'s involving l.s. planes.
Refinement. Refinement of *F*^2^ against ALL reflections. The weighted *R*-factor *wR* and goodness of fit *S* are based on *F*^2^, conventional *R*-factors *R* are based on *F*, with *F* set to zero for negative *F*^2^. The threshold expression of *F*^2^ > σ(*F*^2^) is used only for calculating *R*-factors(gt) *etc*. and is not relevant to the choice of reflections for refinement. *R*-factors based on *F*^2^ are statistically about twice as large as those based on *F*, and *R*- factors based on ALL data will be even larger.

### Fractional atomic coordinates and isotropic or equivalent isotropic displacement parameters (Å^2^)

**Table:**

	*x*	*y*	*z*	*U*_iso_*/*U*_eq_	
O1	0.38761 (7)	0.05350 (5)	0.43124 (5)	0.01571 (11)	
N1	0.60561 (8)	0.01832 (6)	0.38204 (5)	0.01320 (11)	
H1N	0.6179 (16)	−0.0054 (11)	0.4346 (11)	0.022 (3)*	
N2	0.28444 (8)	−0.02275 (6)	0.10021 (5)	0.01491 (11)	
N3	0.31273 (9)	−0.10081 (6)	0.02234 (5)	0.01668 (12)	
N4	0.45718 (9)	−0.10612 (6)	0.05193 (5)	0.01538 (12)	
N5	0.52683 (8)	−0.02989 (5)	0.15250 (5)	0.01189 (10)	
N6	0.60485 (8)	0.20364 (5)	0.29571 (5)	0.01251 (11)	
C1	0.48112 (9)	0.05928 (6)	0.37255 (6)	0.01170 (11)	
C2	0.45700 (9)	0.11124 (6)	0.28435 (6)	0.01159 (11)	
H2	0.3595 (15)	0.1330 (10)	0.2855 (10)	0.017 (3)*	
C3	0.41921 (9)	0.02050 (6)	0.17984 (6)	0.01156 (11)	
C4	0.68763 (9)	−0.01103 (6)	0.21140 (6)	0.01208 (12)	
C5	0.80602 (10)	−0.02132 (7)	0.15637 (7)	0.01617 (13)	
H5	0.7760 (17)	−0.0426 (12)	0.0765 (11)	0.027 (3)*	
C6	0.96362 (10)	−0.00308 (7)	0.21295 (7)	0.01873 (14)	
H6	1.0457 (18)	−0.0093 (12)	0.1781 (12)	0.030 (3)*	
C7	1.00262 (10)	0.02641 (7)	0.32418 (7)	0.01900 (14)	
H7	1.1107 (19)	0.0416 (12)	0.3635 (12)	0.033 (4)*	
C8	0.88362 (9)	0.03591 (7)	0.37831 (7)	0.01614 (13)	
H8	0.9081 (16)	0.0536 (11)	0.4581 (11)	0.022 (3)*	
C9	0.72383 (9)	0.01681 (6)	0.32280 (6)	0.01220 (12)	
C10	0.57954 (10)	0.25511 (7)	0.21242 (6)	0.01617 (13)	
H10A	0.5286 (16)	0.1938 (11)	0.1401 (11)	0.023 (3)*	
H10B	0.4961 (17)	0.2921 (11)	0.2234 (11)	0.025 (3)*	
C11	0.74158 (12)	0.33854 (7)	0.21560 (8)	0.02248 (16)	
H11A	0.8144 (18)	0.2980 (13)	0.1966 (12)	0.033 (4)*	
H11	0.722 (2)	0.3742 (13)	0.1604 (13)	0.040 (4)*	
C12	0.81799 (14)	0.42864 (8)	0.32592 (9)	0.02767 (19)	
H12A	0.753 (2)	0.4792 (14)	0.3395 (13)	0.039 (4)*	
H12B	0.933 (2)	0.4793 (14)	0.3300 (13)	0.039 (4)*	
C13	0.82547 (12)	0.37556 (8)	0.41398 (8)	0.02422 (17)	
H13A	0.9033 (18)	0.3335 (12)	0.4088 (12)	0.031 (4)*	
H13B	0.8589 (17)	0.4330 (12)	0.4846 (11)	0.027 (3)*	
C14	0.65953 (10)	0.28969 (7)	0.40202 (6)	0.01774 (14)	
H14A	0.6707 (17)	0.2514 (11)	0.4561 (11)	0.026 (3)*	
H14B	0.5794 (18)	0.3278 (12)	0.4109 (11)	0.029 (3)*	
O51	0.52486 (10)	0.64389 (6)	0.05346 (5)	0.02384 (13)	
N51	0.40757 (10)	0.50343 (6)	0.11293 (6)	0.01766 (13)	
H51N	0.4212 (19)	0.4560 (13)	0.0574 (13)	0.036 (4)*	
N52	0.67271 (10)	0.77756 (6)	0.37934 (6)	0.02130 (14)	
N53	0.72173 (10)	0.73295 (7)	0.45426 (6)	0.02402 (15)	
N54	0.62468 (10)	0.63061 (7)	0.43334 (6)	0.02147 (14)	
N55	0.50786 (9)	0.60616 (6)	0.34204 (5)	0.01671 (12)	
N56	0.26638 (9)	0.67324 (6)	0.22010 (5)	0.01605 (12)	
C51	0.45755 (11)	0.61293 (7)	0.11881 (6)	0.01729 (14)	
C52	0.43580 (10)	0.69955 (6)	0.21216 (6)	0.01637 (13)	
H52	0.4837 (16)	0.7759 (11)	0.2025 (10)	0.020 (3)*	
C53	0.53997 (10)	0.69786 (7)	0.31100 (6)	0.01685 (13)	
C54	0.38196 (10)	0.49889 (6)	0.29242 (6)	0.01599 (13)	
C55	0.31368 (11)	0.44075 (7)	0.35588 (7)	0.01975 (15)	
H55	0.3508 (16)	0.4735 (11)	0.4336 (11)	0.022 (3)*	
C56	0.19621 (11)	0.33379 (8)	0.30809 (8)	0.02194 (16)	
H56	0.1541 (17)	0.2909 (12)	0.3505 (11)	0.027 (3)*	
C57	0.14557 (11)	0.28606 (7)	0.19694 (8)	0.02214 (16)	
H57	0.0629 (17)	0.2098 (12)	0.1595 (12)	0.029 (3)*	
C58	0.21359 (11)	0.34480 (7)	0.13379 (7)	0.01951 (15)	
H58	0.1781 (17)	0.3104 (12)	0.0555 (11)	0.027 (3)*	
C59	0.33285 (10)	0.45203 (6)	0.18068 (6)	0.01597 (13)	
C60	0.14703 (12)	0.64876 (7)	0.11863 (7)	0.02103 (15)	
H60	0.1632 (16)	0.7177 (11)	0.1003 (11)	0.023 (3)*	
H60B	0.1633 (17)	0.5889 (12)	0.0625 (11)	0.028 (3)*	
C61	−0.02563 (12)	0.60567 (8)	0.13221 (8)	0.02450 (17)	
H61A	−0.1061 (19)	0.5883 (13)	0.0638 (13)	0.038 (4)*	
H61B	−0.0431 (17)	0.5330 (12)	0.1488 (11)	0.027 (3)*	
C62	−0.05395 (13)	0.69164 (9)	0.22197 (9)	0.0310 (2)	
H62A	−0.059 (2)	0.7574 (15)	0.1989 (14)	0.049 (5)*	
H62B	−0.164 (2)	0.6604 (14)	0.2299 (13)	0.039 (4)*	
C63	0.08227 (13)	0.73000 (9)	0.32478 (8)	0.02806 (19)	
H63A	0.075 (2)	0.6703 (14)	0.3558 (13)	0.039 (4)*	
H63B	0.0736 (19)	0.7914 (13)	0.3791 (13)	0.038 (4)*	
C64	0.25074 (11)	0.76573 (7)	0.30339 (7)	0.02088 (15)	
H64A	0.3370 (17)	0.7863 (11)	0.3681 (11)	0.025 (3)*	
H64B	0.2700 (17)	0.8337 (12)	0.2788 (11)	0.030 (3)*	

### Atomic displacement parameters (Å^2^)

**Table:**

	*U*^11^	*U*^22^	*U*^33^	*U*^12^	*U*^13^	*U*^23^
O1	0.0141 (2)	0.0241 (3)	0.0131 (2)	0.0092 (2)	0.00717 (19)	0.0080 (2)
N1	0.0118 (2)	0.0199 (3)	0.0118 (2)	0.0077 (2)	0.0050 (2)	0.0080 (2)
N2	0.0124 (3)	0.0189 (3)	0.0118 (3)	0.0044 (2)	0.0018 (2)	0.0046 (2)
N3	0.0167 (3)	0.0181 (3)	0.0123 (3)	0.0043 (2)	0.0021 (2)	0.0034 (2)
N4	0.0179 (3)	0.0152 (3)	0.0108 (2)	0.0049 (2)	0.0028 (2)	0.0023 (2)
N5	0.0120 (2)	0.0138 (2)	0.0102 (2)	0.0050 (2)	0.00305 (19)	0.00371 (19)
N6	0.0132 (3)	0.0130 (2)	0.0109 (2)	0.0040 (2)	0.0037 (2)	0.00355 (19)
C1	0.0102 (3)	0.0149 (3)	0.0102 (3)	0.0048 (2)	0.0032 (2)	0.0038 (2)
C2	0.0109 (3)	0.0146 (3)	0.0103 (3)	0.0054 (2)	0.0035 (2)	0.0042 (2)
C3	0.0108 (3)	0.0142 (3)	0.0107 (3)	0.0047 (2)	0.0035 (2)	0.0050 (2)
C4	0.0105 (3)	0.0147 (3)	0.0127 (3)	0.0056 (2)	0.0041 (2)	0.0053 (2)
C5	0.0159 (3)	0.0203 (3)	0.0176 (3)	0.0096 (3)	0.0090 (3)	0.0084 (3)
C6	0.0142 (3)	0.0241 (4)	0.0242 (4)	0.0101 (3)	0.0104 (3)	0.0109 (3)
C7	0.0113 (3)	0.0238 (4)	0.0247 (4)	0.0079 (3)	0.0053 (3)	0.0099 (3)
C8	0.0114 (3)	0.0208 (3)	0.0168 (3)	0.0065 (2)	0.0026 (2)	0.0070 (3)
C9	0.0103 (3)	0.0150 (3)	0.0134 (3)	0.0055 (2)	0.0044 (2)	0.0059 (2)
C10	0.0185 (3)	0.0160 (3)	0.0159 (3)	0.0063 (3)	0.0051 (3)	0.0076 (2)
C11	0.0251 (4)	0.0194 (3)	0.0221 (4)	0.0027 (3)	0.0097 (3)	0.0090 (3)
C12	0.0297 (5)	0.0174 (3)	0.0284 (4)	−0.0010 (3)	0.0088 (4)	0.0050 (3)
C13	0.0205 (4)	0.0219 (4)	0.0203 (4)	−0.0014 (3)	0.0043 (3)	0.0015 (3)
C14	0.0177 (3)	0.0176 (3)	0.0132 (3)	0.0027 (3)	0.0049 (3)	0.0010 (2)
O51	0.0383 (4)	0.0196 (3)	0.0197 (3)	0.0109 (3)	0.0165 (3)	0.0097 (2)
N51	0.0272 (3)	0.0141 (3)	0.0140 (3)	0.0077 (2)	0.0090 (3)	0.0054 (2)
N52	0.0236 (3)	0.0198 (3)	0.0173 (3)	0.0063 (3)	0.0042 (3)	0.0032 (2)
N53	0.0253 (4)	0.0247 (3)	0.0186 (3)	0.0083 (3)	0.0019 (3)	0.0044 (3)
N54	0.0247 (4)	0.0246 (3)	0.0143 (3)	0.0102 (3)	0.0018 (3)	0.0056 (2)
N55	0.0202 (3)	0.0180 (3)	0.0126 (3)	0.0074 (2)	0.0042 (2)	0.0055 (2)
N56	0.0200 (3)	0.0147 (3)	0.0128 (3)	0.0066 (2)	0.0037 (2)	0.0033 (2)
C51	0.0245 (4)	0.0157 (3)	0.0137 (3)	0.0078 (3)	0.0070 (3)	0.0059 (2)
C52	0.0221 (3)	0.0142 (3)	0.0135 (3)	0.0063 (3)	0.0059 (3)	0.0049 (2)
C53	0.0206 (3)	0.0162 (3)	0.0143 (3)	0.0070 (3)	0.0060 (3)	0.0044 (2)
C54	0.0192 (3)	0.0163 (3)	0.0150 (3)	0.0075 (3)	0.0059 (3)	0.0068 (2)
C55	0.0243 (4)	0.0234 (4)	0.0189 (3)	0.0120 (3)	0.0102 (3)	0.0119 (3)
C56	0.0226 (4)	0.0242 (4)	0.0269 (4)	0.0099 (3)	0.0119 (3)	0.0159 (3)
C57	0.0207 (4)	0.0194 (3)	0.0278 (4)	0.0057 (3)	0.0071 (3)	0.0111 (3)
C58	0.0222 (4)	0.0165 (3)	0.0190 (3)	0.0057 (3)	0.0042 (3)	0.0065 (3)
C59	0.0202 (3)	0.0158 (3)	0.0150 (3)	0.0078 (3)	0.0065 (3)	0.0071 (2)
C60	0.0260 (4)	0.0214 (4)	0.0149 (3)	0.0088 (3)	0.0022 (3)	0.0060 (3)
C61	0.0238 (4)	0.0219 (4)	0.0229 (4)	0.0070 (3)	0.0005 (3)	0.0041 (3)
C62	0.0237 (4)	0.0301 (5)	0.0333 (5)	0.0114 (4)	0.0046 (4)	0.0010 (4)
C63	0.0242 (4)	0.0281 (4)	0.0259 (4)	0.0074 (3)	0.0098 (3)	−0.0006 (3)
C64	0.0229 (4)	0.0161 (3)	0.0193 (4)	0.0058 (3)	0.0058 (3)	−0.0001 (3)

### Geometric parameters (Å, °)

**Table:**

O1—C1	1.2329 (9)	O51—C51	1.2317 (10)
N1—H1N	0.850 (14)	N51—H51N	0.886 (16)
N1—C1	1.3578 (9)	N51—C51	1.3589 (10)
N1—C9	1.4142 (10)	N51—C59	1.4131 (11)
N2—N3	1.3686 (10)	N52—N53	1.3663 (11)
N2—C3	1.3229 (9)	N52—C53	1.3202 (11)
N3—N4	1.2983 (10)	N53—N54	1.2992 (12)
N4—N5	1.3640 (9)	N54—N55	1.3615 (10)
N5—C3	1.3499 (9)	N55—C53	1.3524 (11)
N5—C4	1.4225 (9)	N55—C54	1.4244 (11)
N6—C2	1.4605 (10)	N56—C52	1.4632 (11)
N6—C10	1.4752 (10)	N56—C60	1.4731 (11)
N6—C14	1.4674 (10)	N56—C64	1.4748 (11)
C1—C2	1.5351 (10)	C51—C52	1.5338 (11)
C2—H2	0.982 (12)	C52—H52	1.026 (13)
C2—C3	1.4944 (10)	C52—C53	1.4955 (11)
C4—C5	1.3923 (10)	C54—C55	1.3908 (11)
C4—C9	1.4004 (10)	C54—C59	1.4005 (11)
C5—H5	1.002 (14)	C55—H55	0.974 (13)
C5—C6	1.3897 (11)	C55—C56	1.3904 (13)
C6—H6	0.936 (15)	C56—H56	0.951 (14)
C6—C7	1.3959 (13)	C56—C57	1.3943 (14)
C7—H7	0.947 (15)	C57—H57	0.992 (14)
C7—C8	1.3877 (12)	C57—C58	1.3912 (13)
C8—H8	1.007 (13)	C58—H58	0.983 (14)
C8—C9	1.3988 (10)	C58—C59	1.3982 (12)
C10—H10A	1.014 (13)	C60—H60	0.982 (13)
C10—H10B	1.001 (14)	C60—H60B	0.997 (14)
C10—C11	1.5196 (12)	C60—C61	1.5258 (14)
C11—H11A	0.969 (15)	C61—H61A	0.991 (16)
C11—H11	0.996 (16)	C61—H61B	1.016 (14)
C11—C12	1.5275 (14)	C61—C62	1.5269 (14)
C12—H12A	0.996 (16)	C62—H62A	1.005 (18)
C12—H12B	1.023 (16)	C62—H62B	0.974 (17)
C12—C13	1.5300 (15)	C62—C63	1.5300 (16)
C13—H13A	0.998 (15)	C63—H63A	0.977 (16)
C13—H13B	0.979 (14)	C63—H63B	0.965 (16)
C13—C14	1.5252 (12)	C63—C64	1.5252 (14)
C14—H14A	0.998 (14)	C64—H64A	0.973 (14)
C14—H14B	0.982 (14)	C64—H64B	1.015 (14)
			
H1N—N1—C1	114.3 (9)	H51N—N51—C51	115.8 (10)
H1N—N1—C9	115.7 (9)	H51N—N51—C59	114.1 (10)
C1—N1—C9	129.81 (6)	C51—N51—C59	130.00 (7)
N3—N2—C3	105.55 (6)	N53—N52—C53	105.52 (7)
N2—N3—N4	111.36 (6)	N52—N53—N54	111.29 (7)
N3—N4—N5	106.08 (6)	N53—N54—N55	106.29 (7)
N4—N5—C3	108.10 (6)	N54—N55—C53	107.76 (7)
N4—N5—C4	122.45 (6)	N54—N55—C54	122.40 (7)
C3—N5—C4	129.44 (6)	C53—N55—C54	129.82 (7)
C2—N6—C10	111.02 (6)	C52—N56—C60	113.57 (7)
C2—N6—C14	112.06 (6)	C52—N56—C64	110.57 (6)
C10—N6—C14	109.94 (6)	C60—N56—C64	109.24 (7)
O1—C1—N1	121.56 (7)	O51—C51—N51	121.40 (7)
O1—C1—C2	120.43 (6)	O51—C51—C52	119.90 (7)
N1—C1—C2	118.00 (6)	N51—C51—C52	118.64 (7)
N6—C2—C1	111.27 (6)	N56—C52—C51	113.38 (7)
N6—C2—H2	113.6 (7)	N56—C52—H52	113.6 (7)
N6—C2—C3	109.40 (6)	N56—C52—C53	108.84 (7)
C1—C2—H2	108.1 (7)	C51—C52—H52	105.9 (7)
C1—C2—C3	107.10 (6)	C51—C52—C53	105.63 (6)
H2—C2—C3	107.0 (7)	H52—C52—C53	109.1 (7)
N2—C3—N5	108.91 (6)	N52—C53—N55	109.13 (7)
N2—C3—C2	128.45 (7)	N52—C53—C52	128.94 (8)
N5—C3—C2	122.63 (6)	N55—C53—C52	121.92 (7)
N5—C4—C5	118.89 (7)	N55—C54—C55	119.38 (7)
N5—C4—C9	119.78 (6)	N55—C54—C59	119.52 (7)
C5—C4—C9	121.33 (7)	C55—C54—C59	121.06 (8)
C4—C5—H5	119.5 (8)	C54—C55—H55	120.3 (8)
C4—C5—C6	119.48 (7)	C54—C55—C56	119.71 (8)
H5—C5—C6	121.0 (8)	H55—C55—C56	119.9 (8)
C5—C6—H6	121.3 (9)	C55—C56—H56	120.7 (9)
C5—C6—C7	119.99 (7)	C55—C56—C57	119.95 (8)
H6—C6—C7	118.7 (9)	H56—C56—C57	119.3 (9)
C6—C7—H7	120.5 (9)	C56—C57—H57	122.7 (8)
C6—C7—C8	120.13 (7)	C56—C57—C58	120.12 (8)
H7—C7—C8	119.4 (9)	H57—C57—C58	117.2 (8)
C7—C8—H8	121.2 (8)	C57—C58—H58	119.8 (8)
C7—C8—C9	120.79 (7)	C57—C58—C59	120.60 (8)
H8—C8—C9	118.0 (8)	H58—C58—C59	119.6 (8)
N1—C9—C4	123.90 (6)	N51—C59—C54	123.27 (7)
N1—C9—C8	117.64 (7)	N51—C59—C58	117.95 (7)
C4—C9—C8	118.27 (7)	C54—C59—C58	118.55 (7)
N6—C10—H10A	109.2 (7)	N56—C60—H60	109.4 (8)
N6—C10—H10B	109.5 (8)	N56—C60—H60B	108.1 (8)
N6—C10—C11	109.81 (7)	N56—C60—C61	108.76 (7)
H10A—C10—H10B	105.0 (11)	H60—C60—H60B	111.6 (11)
H10A—C10—C11	111.8 (8)	H60—C60—C61	109.8 (8)
H10B—C10—C11	111.4 (8)	H60B—C60—C61	109.2 (8)
C10—C11—H11A	108.7 (9)	C60—C61—H61A	108.9 (9)
C10—C11—H11	108.8 (9)	C60—C61—H61B	109.5 (8)
C10—C11—C12	110.94 (8)	C60—C61—C62	111.39 (8)
H11A—C11—H11	107.3 (13)	H61A—C61—H61B	107.4 (12)
H11A—C11—C12	111.0 (9)	H61A—C61—C62	110.2 (9)
H11—C11—C12	109.9 (9)	H61B—C61—C62	109.4 (8)
C11—C12—H12A	110.7 (9)	C61—C62—H62A	109.2 (10)
C11—C12—H12B	110.5 (9)	C61—C62—H62B	109.1 (9)
C11—C12—C13	110.97 (8)	C61—C62—C63	110.61 (8)
H12A—C12—H12B	105.6 (13)	H62A—C62—H62B	102.9 (13)
H12A—C12—C13	108.5 (9)	H62A—C62—C63	110.7 (10)
H12B—C12—C13	110.4 (9)	H62B—C62—C63	114.0 (9)
C12—C13—H13A	110.2 (9)	C62—C63—H63A	111.7 (10)
C12—C13—H13B	110.7 (8)	C62—C63—H63B	111.2 (9)
C12—C13—C14	111.35 (8)	C62—C63—C64	110.88 (9)
H13A—C13—H13B	110.0 (12)	H63A—C63—H63B	105.8 (13)
H13A—C13—C14	106.0 (8)	H63A—C63—C64	107.8 (9)
H13B—C13—C14	108.4 (8)	H63B—C63—C64	109.4 (9)
N6—C14—C13	109.53 (7)	N56—C64—C63	110.32 (7)
N6—C14—H14A	107.6 (8)	N56—C64—H64A	108.6 (8)
N6—C14—H14B	109.8 (8)	N56—C64—H64B	108.5 (8)
C13—C14—H14A	109.5 (8)	C63—C64—H64A	110.7 (8)
C13—C14—H14B	109.4 (8)	C63—C64—H64B	111.4 (8)
H14A—C14—H14B	111.0 (12)	H64A—C64—H64B	107.2 (12)
			
C3—N2—N3—N4	0.15 (9)	C53—N52—N53—N54	0.19 (10)
N2—N3—N4—N5	0.29 (8)	N52—N53—N54—N55	0.05 (10)
N3—N4—N5—C3	−0.62 (8)	N53—N54—N55—C53	−0.27 (9)
N3—N4—N5—C4	−179.48 (7)	N53—N54—N55—C54	177.97 (8)
C9—N1—C1—O1	179.78 (7)	C59—N51—C51—O51	178.22 (9)
C9—N1—C1—C2	1.11 (11)	C59—N51—C51—C52	0.99 (14)
C10—N6—C2—C1	−178.53 (6)	C60—N56—C52—C51	−50.44 (9)
C10—N6—C2—C3	63.32 (7)	C60—N56—C52—C53	−167.67 (6)
C14—N6—C2—C1	−55.17 (8)	C64—N56—C52—C51	−173.65 (7)
C14—N6—C2—C3	−173.31 (6)	C64—N56—C52—C53	69.12 (8)
O1—C1—C2—N6	123.80 (7)	O51—C51—C52—N56	126.51 (9)
O1—C1—C2—C3	−116.68 (7)	O51—C51—C52—C53	−114.40 (9)
N1—C1—C2—N6	−57.52 (8)	N51—C51—C52—N56	−56.22 (10)
N1—C1—C2—C3	62.01 (8)	N51—C51—C52—C53	62.87 (10)
N3—N2—C3—N5	−0.53 (8)	N53—N52—C53—N55	−0.36 (9)
N3—N2—C3—C2	178.55 (7)	N53—N52—C53—C52	−179.19 (8)
N4—N5—C3—N2	0.73 (8)	N54—N55—C53—N52	0.40 (9)
N4—N5—C3—C2	−178.43 (6)	N54—N55—C53—C52	179.32 (7)
C4—N5—C3—N2	179.49 (7)	C54—N55—C53—N52	−177.66 (8)
C4—N5—C3—C2	0.33 (12)	C54—N55—C53—C52	1.26 (13)
N6—C2—C3—N2	−121.66 (8)	N56—C52—C53—N52	−124.11 (9)
N6—C2—C3—N5	57.31 (9)	N56—C52—C53—N55	57.19 (10)
C1—C2—C3—N2	117.62 (8)	C51—C52—C53—N52	113.82 (9)
C1—C2—C3—N5	−63.41 (9)	C51—C52—C53—N55	−64.87 (10)
N4—N5—C4—C5	33.23 (10)	N54—N55—C54—C55	36.85 (11)
N4—N5—C4—C9	−146.46 (7)	N54—N55—C54—C59	−140.99 (8)
C3—N5—C4—C5	−145.37 (8)	C53—N55—C54—C55	−145.34 (9)
C3—N5—C4—C9	34.94 (11)	C53—N55—C54—C59	36.82 (12)
N5—C4—C5—C6	179.98 (7)	N55—C54—C55—C56	−177.01 (8)
C9—C4—C5—C6	−0.34 (12)	C59—C54—C55—C56	0.80 (13)
C4—C5—C6—C7	−0.64 (12)	C54—C55—C56—C57	−0.99 (13)
C5—C6—C7—C8	0.98 (13)	C55—C56—C57—C58	0.65 (14)
C6—C7—C8—C9	−0.34 (13)	C56—C57—C58—C59	−0.11 (14)
C7—C8—C9—N1	174.59 (7)	C57—C58—C59—N51	174.60 (8)
C7—C8—C9—C4	−0.62 (11)	C57—C58—C59—C54	−0.09 (13)
N5—C4—C9—N1	5.76 (11)	N55—C54—C59—N51	3.16 (12)
N5—C4—C9—C8	−179.36 (7)	N55—C54—C59—C58	177.55 (7)
C5—C4—C9—N1	−173.92 (7)	C55—C54—C59—N51	−174.65 (8)
C5—C4—C9—C8	0.96 (11)	C55—C54—C59—C58	−0.26 (12)
C1—N1—C9—C4	−43.24 (12)	C51—N51—C59—C54	−41.97 (13)
C1—N1—C9—C8	141.84 (8)	C51—N51—C59—C58	143.61 (9)
C2—N6—C10—C11	−171.45 (6)	C52—N56—C60—C61	171.56 (7)
C14—N6—C10—C11	63.98 (8)	C64—N56—C60—C61	−64.50 (9)
N6—C10—C11—C12	−57.34 (10)	N56—C60—C61—C62	58.95 (10)
C10—C11—C12—C13	50.96 (11)	C60—C61—C62—C63	−52.02 (12)
C11—C12—C13—C14	−50.81 (11)	C61—C62—C63—C64	50.42 (12)
C2—N6—C14—C13	172.62 (7)	C52—N56—C64—C63	−170.26 (8)
C10—N6—C14—C13	−63.41 (9)	C60—N56—C64—C63	64.06 (10)
C12—C13—C14—N6	56.84 (10)	C62—C63—C64—N56	−56.70 (11)

### Hydrogen-bond geometry (Å, °)

**Table:**

*D*—H···*A*	*D*—H	H···*A*	*D*···*A*	*D*—H···*A*
N1—H1N···O1^i^	0.850 (14)	2.069 (14)	2.9089 (9)	169.4 (13)
N51—H51N···O51^ii^	0.886 (16)	1.929 (16)	2.8116 (10)	173.6 (14)
C6—H6···N2^iii^	0.936 (15)	2.531 (15)	3.4638 (11)	174.8 (12)
C7—H7···O1^iii^	0.947 (15)	2.406 (15)	3.3394 (10)	168.4 (13)
C55—H55···N54^iv^	0.974 (13)	2.548 (13)	3.2293 (11)	127.0 (10)

Symmetry codes: (i) −*x*+1, −*y*, −*z*+1; (ii) −*x*+1, −*y*+1, −*z*; (iii) *x*+1, *y*, *z*; (iv) −*x*+1, −*y*+1, −*z*+1.
